# Comparison of novel rapid diagnostic of blood culture identification and antimicrobial susceptibility testing by Accelerate Pheno system and BioFire FilmArray Blood Culture Identification and BioFire FilmArray Blood Culture Identification 2 panels

**DOI:** 10.1186/s12866-021-02403-y

**Published:** 2021-12-18

**Authors:** Dorothy T. T. Sze, Candy C. Y. Lau, Tsz-Ming Chan, Edmond S. K. Ma, Bone S. F. Tang

**Affiliations:** 1 Department of Pathology, Hong Kong Sanatorium and Hospital, Hong Kong, Special Administrative Region China; 2 Department of Pathology, Hong Kong Sanatorium and Hospital, Hong Kong, Special Administrative Region China

**Keywords:** Susceptibility testing, Pheno, Blood culture, Antimicrobial resistance, Identification, Rapid tests, Bloodstream infections, Filmarray

## Abstract

**Background:**

Conventional turnaround time (TAT) for positive blood culture (PBC) identification (ID) and antimicrobial susceptibility testing (AST) is 2–3 days. We evaluated the TAT and ID/AST performance using clinical and seeded samples directly from PBC bottles with different commercial approaches: (1) Accelerate Pheno® system (Pheno) for ID/AST; (2) BioFire® FilmArray® Blood Culture Identification (BCID) Panel and/ or BCID2 for ID; (3) direct AST by VITEK® 2 (direct AST); and (4) overnight culture using VITEK® 2 colony AST.

**Results:**

A total of 141 PBC samples were included in this evaluation. Using MALDI-TOF (Bruker MALDI Biotyper) as the reference method for ID, the overall monomicrobial ID sensitivity/specificity are as follows: Pheno 97.9/99.9%; BCID 100/100%; and BCID2 100/100%, respectively. For AST performance, broth microdilution (BMD) was used as the reference method. For gram-negatives, overall categorical and essential agreements (CA/EA) for each method were: Pheno 90.3/93.2%; direct AST 92.6/88.5%; colony AST 94.4/89.5%, respectively. For gram-positives, the overall CA/EAs were as follows: Pheno 97.2/98.89%; direct AST 97.2/100%; colony AST 97.2/100%, respectively. The BCID/BCID2 and direct AST TATs were around 9–20 h (1/9-19 h for ID with resistance markers/AST), with 15 min/sample hands-on time. In comparison, Pheno TATs were around 8–10 h (1.5/7 h for ID/AST) with 2 min/sample hands-on time, maintains a clinically relevant fast report of antibiotic minimal inhibitory concentration (MIC) and allows for less TAT and hands-on time.

**Conclusion:**

In conclusion, to the best of our knowledge, this is the first study conducted as such in Asia; all studied approaches achieved satisfactory performance, factors such as TAT, panel of antibiotics choices and hands-on time should be considered for the selection of appropriate rapid ID and AST of PBC methods in different laboratory settings.

**Supplementary Information:**

The online version contains supplementary material available at 10.1186/s12866-021-02403-y.

## Introduction

Bloodstream infections (BSI) are associated with significant morbidity and mortality, with rates up to 60% reported depending on various factors [1]. Recent data from the SENTRY surveillance program indicate the most frequent pathogens for BSI are *Escherichia coli*, *Staphylococcus aureus*, and *Klebsiella pneumoniae* in the Asia-Pacific region [2]. Delays in the diagnosis and the following appropriate antimicrobial treatment plans are associated with increased mortality rate and poor clinical outcomes [3]. To provide earlier ID and AST results for pathogens responsible for BSI is important for clinicians to escalate or deescalate antimicrobial treatment for patients.

Conventionally, PBC are subcultured to obtain pure colony growth, which usually takes 24–48 h. Methods such as Matrix-Assisted Laser Desorption Ionization Time-of-Flight Mass Spectrometry (MALDI-TOF MS), manual biochemical tests, or automated ID systems, such as VITEK® 2 (bioMérieux SA, Marcy-l’Étoile, France), are routinely used for ID from isolated colony, while AST is typically performed using Kirby-Bauer disk diffusion, Epsilometer test (E test, bioMérieux) or automated AST systems, such as VITEK® 2. Generally, the results from these traditional methods are available after 2 to 3 days.

In recent years, time to ID from blood culture has been shortened by using newer methods such as extracting pathogens directly from PBC with sample preparation kits such as Sepsityper (Bruker Daltonik GmbH, Bremen, Germany), short-term culture followed by MALDI-TOF MS [4], by using multiplexed PCR molecular detection, such as FilmArray BCID (BioFire, Salt Lake City, UT), Verigene Blood culture system (Luminex®) or ePlex BCID panels (GenMark Dx®) [5]. The time to result of BCID is around 1 h with 2 min of hands-on time, which is significantly shorter than those in routine ID methods [6]. BCID2 has expanded to 15 additional target groups and seven antimicrobial resistance (AMR) genes when compared to the BCID Panel [7]*.* These molecular methods however do not generate phenotypic antimicrobial results, such as MICs.

Despite the improvement in ID TAT, until recently AST still relied on colony-based methods, which requires overnight incubation. The European Committee on Antimicrobial Susceptibility Testing (EUCAST) has published a rapid AST (RAST) method directly from PBC using disk diffusion [8] and the Clinical Laboratory Standards Institute (CLSI) has recently approved a direct from blood culture disk diffusion method to be published in the M100 31st edition (2020 Summer Subcommittee on AST Meeting, accessed online unpublished).

The Accelerate Pheno® system (Accelerate Diagnostics, Inc., Tucson, AZ) is a closed, self-contained, and fully automated system providing ID and AST results direct from PBC. It provides ID within 90 min and AST in around 7 h. Bacterial cell extraction is automatically performed using gel electrofiltration, where an electric field is applied causing lysis of blood cells and/or other sample debris that then pass into the gel wall while bacterial/yeast cells remain intact. Fluorescence in situ hybridization probes are used to identify genus and species-specific bacteria and yeast, covering 17 target microorganisms. Once the ID result is available, AST is performed (on AST-eligible bacteria) using the morphokinetic cellular analysis technology to measure distinct morphokinetic features of live microbial cells responding to antimicrobials to generate MICs and susceptibility results [9, 10].

In this study, ID/AST performance, the average time to results, and workflow were compared between (1) Pheno for both ID and AST and (2) BCID and / or BCID2 Panel for ID in conjunction with direct AST when compared to conventional colony ID and AST. We also evaluated an in-house saponin extraction method based on the modification of the VITEK® Mass Spectrometry procedure for direct AST [11]. Previously published studies have focused on ID performance or utilized more complex procedures including filtration and lysis based methods [12]. These published techniques also have some drawbacks such as the requirements of sophisticated equipment such as 4 °C centrifuge [13] or serum separator tubes [14–17], increased hands-on time [18], and relatively low identification accuracy [17]. The reference method used as a comparator for all experimental methods was standard Bruker MALDI-TOF MS Biotyper system from colony growth for ID and BMD for AST.

## Methods and materials

### Clinical specimens

A total of 74 fresh, patient data de-identified, residual PBC samples collected from hospitalized patients in Hong Kong Sanatorium & Hospital during the period from Aug 2019 to Feb 2020 were included in this study. These PBC samples were obtained from BACTEC Plus Aerobic/F Medium, BACTEC Peds Plus/F Medium and BACTEC Lytic/10 Anaerobic/F Medium bottles (Becton Dickinson Diagnostic Systems, Sparks, MD). Specimens were enrolled in the study if they were within 8 h of positivity. Specimens exceeding 8 h positivity, collected from the same patient with same organism identified in previously enrolled patients, or samples with gram stain morphology not representative of organisms on the Accelerate PhenoTest**®** BC kit (such as gram positive rods) were excluded from the study. Enrolment of clinical samples for BCID/BCID2 were based on the manufacturer’s package inserts. Due to the time difference in the installations of comparative systems in our institution, only part of the clinical samples were evaluated for all the comparative systems. All specimens were subcultured on to trypticase soy agar with 5% sheep’s blood (SBA) to perform colony-based ID and AST as described below.

### Bacterial and fungal isolates selection and screening for seeding studies

Archived fungal and bacterial isolates with known antimicrobial susceptibility profiles or resistance gene profiles were included in this study. The AST profiles were determined by Kirby-Bauer disc diffusion using BD antimicrobial discs (BD Diagnostic Systems, Sparks, MD) or by E-test (Oxoid, Hampshire, United Kingdom). Gram-negative carbapenem resistance gene profiles were determined by the Carba 5 assay (NG biotect, Guipry, France) for carbapenemase genes (*bla*_KPC_ *, bla*_VIM_ *, bla*_NDM-_, *bla*_OXA-48_ and *bla*_IMP_), which were further confirmed by PCR in the Department of Health, Hong Kong Special Administrative Region, a local reference laboratory. Some isolates possessing the colistin-resistance *mcr-1* gene as detected by polymerase chain reaction (PCR) were also included.

Phenotypic expression of extended spectrum beta lactamase (ESBL) production in Enterobacterales was detected using disc diffusion by applying cephalosporin discs (ceftazidime and cefotaxime) with and without clavulanic acid where an increase in zone diameter ≥ 5 mm for the agent tested in combination with clavulanic acid versus the zone diameter of the agent tested alone confirms ESBL production [19] While not a validated method for genotypic resistance detection from isolate, some isolates were further tested by off-label use of BioFire® FilmArray® Pneumonia Panel which contains *bla*_CTX-M_ as a detection target (BioFire, Salt Lake City, UT) as a confirmatory method for BCID2 *bla*_CTX-M_ [20].

**Fig. 1:**
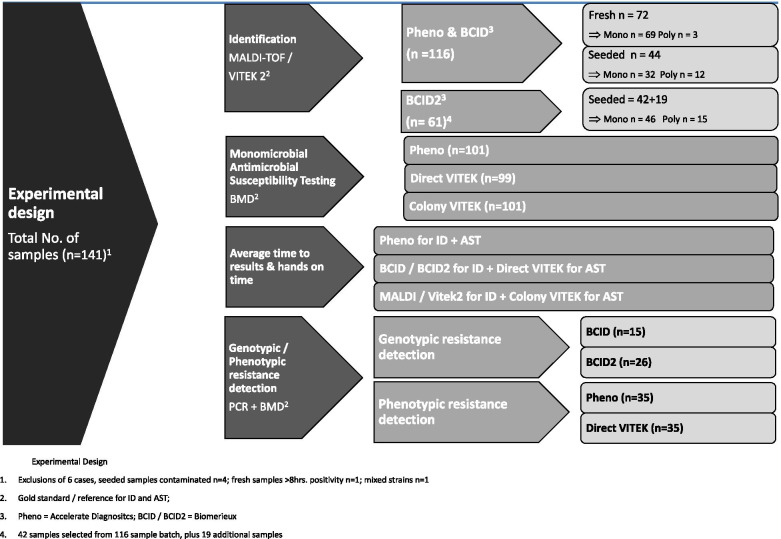
Experimental Design. 1. Exclusions of 6 cases, seeded samples contaminated *n* = 4; fresh samples > 8 h. positivity *n* = 1; mixed strains *n* = 1. 2. Gold standard / reference for ID and AST; 3. Pheno = Accelerate Diagnositcs; BCID / BCID2 = Biomerieux. 4. Forty-two samples selected from 116 sample batch, plus 19 additional samples

Gram-positive resistance gene profiles were determined by testing for vancomycin resistance genes (*vanA*/*B*) and methicillin resistance gene (*mecA*) by GeneXpert vanA/vanB assay and MRSA assay (Cepheid, Sunnyvale, CA), respectively. The experimental design is shown in Fig. 1.

### Preparation of seeded blood cultures

Archived bacterial and fungal isolates were grown on SBA and Sabouraud agar, respectively at 37 °C for 18–24 h prior to use for seeded studies. A suspension with a turbidity of 0.5 McFarland or 2.0 McFarland was prepared from fresh bacterial or fungal colonies, respectively. The suspension was serially diluted and then 1 ml of this 10^6^-fold diluted suspension (~ 100 cells/mL) was inoculated into a BACTEC Plus Aerobic/F Medium BC bottle containing 8–10 ml fresh human donor blood. The seeded BC bottle was incubated in BD BACTEC FX continuous monitoring blood culture system. BC bottles that flagged positive and could be tested within 8 h positivity were included in this study. All positively seeded BC were subcultured to SBA to perform colony-based ID and AST as described below. A total of 67 blood cultures were seeded.

### Identification of bacterial and fungal isolates

ID of isolates was confirmed by Bruker MALDI Biotyper (Bruker, Bremen, Germany) and Vitek 2 Compact system (bioMérieux SA, Marcy-l’Étoile, France) on colony growth from the PBC bottle as previously described [21–23]. Briefly, bacterial and fungal isolates grown on SBA at 37 °C for 24–48 h. Samples were spotted onto the target plate, overlaid with 1 μl matrix solution (Bruker α-cyano), and analysed by the Bruker MALDI Biotyper system. Fungal isolates were subjected to ethanol-formic acid extraction protocol according to the manufacturers’ instructions prior to being spotted on the plate. Spectra were analysed with MALDI MBT compass version 4.1.80 and Reference Library DB-7854 (version H, Bruker). Scores ≥2.0 were considered acceptable.

ID of bacterial isolates was also performed on Vitek 2 Compact system. Briefly, a bacterial suspension with turbidity of 0.5 McFarland using 0.45% sterile saline according to manufacturer’s instructions was prepared. The inoculum was subsequently tested with Vitek 2 GP67 and Vitek 2 GN69 and XN06 on the Vitek 2 Compact system.

### Accelerate PhenoTest® BC kit

Accelerate PhenoTest*®* BC kit testing was performed using the Accelerate Pheno™ system according to the manufacturer’s instructions and as previously described [9]. Briefly, the PBC bottle was vortexed at 3000 rpm for 10 s and 500 μl of PBC broth was collected using a syringe with 21-gauge needle inside a Class II biosafety cabinet and loaded into the Accelerate PhenoTest*®* BC kit sample vial. The sample vial was placed in the reagent cartridge before loading into the Accelerate Pheno*®* system along with a test cassette. The Accelerate PhenoTest*®* BC kit was tested with CE-IVD software version 1.4.1.28 with 2019 CLSI breakpoints, except for colistin for which ≤2 was considered susceptible and daptomycin for which there was no resistant breakpoint.

### Vitek direct AST

PBC bottle was vortexed and 1 ml of PBC broth was collected. Two hundred microliters of 5% saponin solution (Sigma-Aldrich, St. Louis, USA) were added into the PBC broth, mixed thoroughly and incubated at room temperature for 5 mins. The tube was centrifuged at 13,000 rpm for 2 mins, and the supernatant was discarded. The pellet was washed with 1 ml-distilled water and centrifuged at 13,000 rpm for 1 min and the supernatant was discarded. The pellet was washed twice with 1 ml 0.45% sterile saline and the supernatant was discarded after centrifugation. The pellet was resuspended in 30–50 μl of 0.45% sterile saline. This suspension was used for preparing the inoculum for AST according to Vitek colony AST procedures. Ten microliters of the inoculum were inoculated onto SBA for purity check.

### Vitek Colony AST

Vitek 2 AST-GN69 and XN06 cards were used for Gram-negative organisms while GP-67 was used for Gram-positive organisms. Overnight bacterial and fungal isolates were used for preparing a suspension with turbidity of 0.5 McFarland using 0.45% sterile saline, according to manufacturer’s instructions. A total of 145 μl of this inoculum was transferred to another 2 tubes containing 3 ml 0.45% sterile saline each and subsequently used in AST on Vitek 2 Compact system. AST results were reported in 6–18 h depending on the growth rate. Ten microliters of the inoculum were inoculated onto SBA for purity check.

### Broth microdilution

Frozen reference BMD was performed in triplicate on all bacterial isolates at Accelerate Diagnostics, Inc. (Tucson, Arizona) according to CLSI standards, as previously described [9]. The modal MIC was used as the reference BMD result and if no mode was obtained for a drug, BMD was repeated in triplicate, using the mode of all 6 results as the reference BMD result.

### FilmArray blood culture identification

BioFire® FilmArray® BCID and BCID 2 panel were used for identifying organism(s) present in the BC according to manufacturer’s instructions. BCID and BCID2 utilize the same operational procedure. Briefly, 200 μl of samples were collected from a PBC and lysed with 500 μl Sample Dilution Buffer in a provided sample vial. This sample vial was injected into a BCID/BCID2 pouch pre-rehydrated with Hydration Solution. The pouch was inserted into FilmArray system to start the identification.

This study was conducted in full accordance with the principles of the Declaration of Helsinki. Samples were taken as part of the standard patient care and used anonymously.

#### Statistical analysis

For performance analysis, Bruker MALDI Biotyper from colony and BMD results were used as the gold standard/reference for ID and AST respectively. To evaluate the ID concordance between MALDI with Pheno and BCID/BCID2, sensitivity and specificity, were calculated. For comparable analysis, microorganisms such as *Staphylococcus*, *Enterococcus* and *Streptococcus,* where results from MALDI matched results with Pheno and BCID/BCID2 only on genus level, the results would also be regarded as correct identifications.

For AST performance analysis, MIC was measured and categorized into susceptible (S), intermediate (I), or resistant (R) using 2019 CLSI breakpoints. Results from BMD and all three AST methods (Pheno, Vitek direct AST, Vitek colony AST) were truncated to the same range for comparable analysis. Only isolates for which there were results from all 3 AST methods were included in analysis. Agreement and discrepancies between the methods were classified as follows: categorical agreement (CA), essential agreement (EA), minor error (mE), major error (ME) and very major error (VME). Categorical agreement was calculated as the percentage of S, or I, or R of the investigational method (Pheno or direct AST) that matched BMD results. Essential agreement referred to the percentage of MIC results that fell within one-doubling dilution when compared to the reference method. Minor error was defined as the investigational method showed intermediate result while the reference method showed either susceptible or resistant results or vice versa (susceptible/resistant versus intermediate susceptibility). Major error was defined when the investigational method result was resistant while the reference method was susceptible (false resistance). Very major error referred to results that were tested as susceptible by the investigational method but resistant by the reference method (false susceptibility).

## Results

In total, 141 samples (74 fresh clinical and 67 seeded samples) were enrolled for this study over a 6-month period at the Hong Kong Sanatorium and Hospital. Six samples (*n* = 4 seeded samples contaminated by other organisms and fresh samples *n* = 1 > 8 h of positivity, and *n* = 1 for mixed strains) were excluded. One hundred and sixteen samples were evaluated by Pheno and BCID, including 42 samples evaluated with BCID2 as well. Of the 116 evaluated, 101 were monomicrobial (69 fresh samples, 32 seeded samples) and 15 were polymicrobial samples (3 fresh, 12 seeded). One hundred and one samples were evaluated for the different ST methods except in two cases for direct AST due to modified experimental design at a later stage (supplementary table). Additional 19 samples were tested by BCID2 only, making it 61 samples in total for BCID2 evaluation. Of the 61 samples, 46 are monomicrobial with the following targets detected; *Klebsiella* species (*n* = 10), *E. coli* (*n* = 6), *Pseudomonas aeruginosa* (*n* = 4), *Acinetobacter baumannii* (*n* = 3), *Coagulase-negative Staphylococcus* species (*n* = 3), *Staphylococcus* species (*n* = 2), *Enterococcus faecium* (*n* = 2), *Enterobacter* species (*n* = 2), *Staphylococcus lugdunensis* (*n* = 1), *Streptococcus pyogenes* (*n* = 1), *Streptococcus pneumoniae* (*n* = 1), *Streptococcus agalactiae* (*n* = 1), *Streptococcus dysgalactiae* (*n* = 1), *Enterococcus faecalis* (*n* = 1), *Serratia marcescens* (*n* = 1), *Citrobacter* species (*n* = 1), *Salmonella* (*n* = 1), *Neisseria meningitidis* (*n* = 1), *Bacteroides fragilis* (*n* = 1), *Candida auris* (*n* = 1), *Cryptococcus neoformans* (*n* = 1), no targets detected (*n* = 1), and the remaining (*n* = 15) were polymicrobials.

### Identification

The identification results were compared to the reference results of MALDI-TOF (Tables 1 and 2). Organisms that were correctly identified on the genus level probes would be defined as true positive. The correct identification rate of Pheno, BCID and BCID2 for gram-positive bacteria was 94.6% (35/37), 100% (37/37) and 100% (13/13) respectively, for gram-negative bacteria was 98.3% (59/60), 100% (60/60) and 100% (29/29) respectively. All the yeasts were correctly identified by the three systems. These results exclude polymicrobial infections.

**Table 1:** Identification performance of gram-positives, gram-negatives and yeast by Pheno and BCID compared to MALDI-TOF MS

		Accelerate Pheno						BCID					
Probes detected	# of isolates	TP	FN	TN	FP	Sensitivity (%)	Specificity (%)	TP	FN	TN	FP	Sensitivity (%)	Specificity (%)
**Gram Positive**													
***S. aureus***	5	5	0	96	0	–	–	5	0	96	0	–	–
***S. lugdunesis***	2	2	0	99	0	–	–	2	0	99	0	–	–
**Coagulase-negative** ***Staphylococcus*** **spp.**	11	11	0	90	0	100.0	100.0	11	0	90	0	100.0	100.0
***E. faecium***	4	3	0	97	1	–	–	4	0	97	0	–	–
***E. faecalis***	7	6	1	94	0	–	–	7	0	94	0	–	–
***Streptococcus*** **species** ^a^	8	8	0	93	0	–	–	8	0	93	0	–	–
**Gram Positive Total**	**37**	**35**	**1**	**569**	**1**	**97.2**	**99.8**	**37**	**0**	**569**	**0**	**100.0**	**100.0**
**Gram Negative**													
***E. coli***	28	27	1	73	0	96.4	100.0	28	0	73	0	100.0	100.0
***Klebsiella*** **species**	15	15	0	86	0	100.0	100.0	15	0	86	0	100.0	100.0
***Enterobacter*** **species**	1	1	0	100	0	–	–	1	0	100	0	–	–
***Citrobacter*** **species**	3	3	0	98	0	–	–	3	0	98	0	–	–
***S. marcescens***	1	1	0	100	0	–	–	1	0	100	0	–	–
***P. aeruginosa*** ^b^	8	7	0	95	0	–	–	7	0	95	0	–	–
***A. baumannii***	4	4	0	97	0	–	–	4	0	97	0	–	–
**Gram Negative Total**	**60**	**58**	**1**	**649**	**0**	**98.3**	**100**	**59**	**0**	**649**	**0**	**100.0**	**100.0**
**Anaerobe Total** ^c^	**1**	**0**	**0**	**102**	**0**	**–**	**–**	**0**	**0**	**101**	**0**	**N/A**	**N/A**
**Yeast**													
***C. albicans***	2	2	0	101	0	–	–	2	0	98	0	–	–
***C. tropicalis***	1	0	0	102	0	–	–	1	0	100	0	–	–
**Yeast Total**	**3**	**2**	**0**	**101**	**0**	**–**	**–**	**3**	**0**	**98**	**0**	–	–
**Total no. of Samples**	**101**	**2**	**0**	**1421**	**0**	**97.9**	**99.9**	**99**	**0**	**1417**	**0**	**100.0**	**100.0**

^a^ *Streptococcus* species: *S.s pyogenes* (*n* = 2), *S. agalactiae* (*n* = 2), *S. dysgalactiae* (*n* = 1), *S. pneumoniae* (*n* = 1), *S. mitis/oralis* (*n* = 1), *S. gordonii* (*n* = 1)

^b^ *P. aeruginosa*: *P. aeruginosa* (*n* = 7), *P. nitroreducens* (*n* = 1)

^c^Anaerobe: *B. thetaiotaomicron* (*n* = 1)

**Table 2:** Identification performance of gram-positives, gram-negatives and yeast by BCID2 compared to MALDI-TOF MS

Probes	# of isolates	TP	FN	TN	FP	Sensitivity (%)	Specificity (%)
**Gram Positive**							
***S. aureus***	2	2	0	44	0	–	–
***S. lugdunesis***	1	1	0	45	0	–	–
**Coagulase-negative** ***Staphylococcus*** **species**	3	3	0	43	0	–	–
***E. faecium***	2	2	0	44	0	–	–
***E. faecalis***	1	1	0	45	0	–	–
***Streptococcus*** **species**	4	4	0	42	0	–	–
**Gram Positive Total**	**13**	**13**	0	**263**	**0**	**100.0**	**100.0**
**Gram Negative**							
***E. coli***	6	6	0	40	0	–	–
***Klebsiella*** **species**	10	10	0	36	0	100.0	100.0
***Enterobacter*** **species**	2	2	0	44	0	–	–
***Citrobacter*** **species**	1	1	0	45	0	–	–
***S. marcescens***	1	1	0	45	0	–	–
***P. aeruginosa***	4	4	0	42	0	–	–
***A. baumannii***	3	3	0	43	0	–	–
***Salmonella***	1	1	0	45	0	–	–
***N. meningitidis***	1	1	0	45	0	–	–
**Gram Negative Total**	**29**	**29**	**0**	**385**	**0**	**100.0**	**100.0**
**Anaerobes Total**	1	1	0	16	0	–	–
**Yeasts**						–	–
***C. albicans***	1	1	0	0	0	–	–
***C. neoformans***	1	1	0	0	0	–	–
**Yeast Total**	**2**	**2**	**0**	**44**	**0**	–	–
**No organisms Total**	**1**	**0**	**0**	**46**	**0**	–	–
**Total no. of Samples**	**46**	**74**	**0**	**754**	**0**	**100.0**	**100.0**

For Pheno, 1 false negative *E. faecalis* and 1 false positive *E. faecium* (*E. gallinarum* by reference method) were observed. Additionally, 3 off-panel organisms were included in the evaluation (*Bacteroides thetaiotaomicron, Candida tropicalis* and *Pseudomonas nitroreducens*) and were correctly resulted as negative for all on-panel targets.

For BCID, 2 off-panel organisms *(B. thetaiotaomicron* and *P. nitroreducens*) were also reported as negative for all on-panel targets.

For BCID2, 1 case with no organism growth on subculture was included in the evaluation and tested negative. Noteworthy, *Citrobacter freundii* is not available on the species-specific BCID2 panel but was detected positive as Enterobacterales group of organisms.

As shown in Table 3, of the 15 polymicrobial samples, 13 cases were mixed with 2 microorganisms and 2 cases were mixed with 3 microorganisms. Among the 13 cases with 2 organisms detected, Pheno detected 1 out of the 2 microorganisms in 10 cases (77%) and correctly identified both microorganisms were in 2 cases (15.4%). There was 1 case where neither organism in the sample was detected by Pheno. Whereas BCID detected both organisms in 12 cases (92.3%) (12/13) for BCID. and 1 organism out of the two in 1 case (7.7%). For the 2 cases containing 3 microorganisms, BCID was able to detect all 3 microorganisms in both cases (2/2). For Pheno, only 1 of the 3 microorganisms was detected in both cases. As for BCID2, of all 15 polymicrobials, all on-panel organisms were correctly identified, including those cases (*n* = 3) of on-panel organisms mixed with off-panel organism with no false positive results; 1) *Proteus hauseri* mixed with *Enterococcus casseliflavus* were detected as *Proteus* species, 2) *Burkholderia cepacia complex* mixed with *Pseudomonas aeruginosa* were detected as *Pseudomonas aeruginosa*, and 3) *Klebsiella pneumoniae* mixed with *Enterobacter cloacae complex* and *Morganella morganii* were detected as *Klebsiella* species and *Enterobacter cloacae complex*).

**Table 3:** Overview of polymicrobial identification results and the availability of AST on Pheno system

Culture ID	# of organisms detected	Accelerate ID	BCID	Accelerate AST reported
**Fresh Polymicrobial**				
*E. coli*	3	*E. coli*	*E. coli*	*E. coli*
*K. aerogenes*			*Enterobacteriaceae*	
*P. hauseri*			*Proteus* species	
*P. hauseri*	2	*E. faecium*	*Enterococcus*	Not available
*E. casseliflavus*		*Proteus* species	*Proteus* species	
*S. epidermidis*	2	*No results*	*Staphylococcus* species	Not available
*C. tropicalis*			*C. tropicalis*	
**Seeded Polymicrobial**				
*K. pneumoniae*	2	*Klebsiella* species	*K. pneumoniae*	*Klebsiella* species
*S. aureus*			*S. aureus*	
*E. coli*	2	*E. coli*	*E. coli*	*E. coli*
*E. faecalis*			*Enterococcus* species	
*K. pneumoniae*	2	*E. faecalis*	*K. pneumoniae*	*E. faecalis*
*E. faecalis*			*Enterococcus* species	
*S. aureus*	2	*S. aureus*	*Staphylococcus* species	*S. aureus*
*S. epidermidis*				
*E. faecium*	2	*E. faecium*	*Enterococcus*	Not available
*S. aureus*		*S. aureus*	*S. aureus*	
*S. marcescens*	2	*S. marcescens*	*S. marcescens*	*S. marcescens*
*C. albicans*			*C. albicans*	
*K. pneumoniae*	2	*C. albicans*	*K. pneumoniae*	Not available
*C. albicans*			*C. albicans*	
*A. baumannii*	2	*A. baumannii*	*A. baumannii*	*A. baumannii*
*C. albicans*			*C. albicans*	
*E. coli*	2	*E. coli*	*E. coli*	*E. coli*
*C. albicans*			*C. albicans*	
*P. aeruginosa*	2	*C. albicans*	*P. aeruginosa*	Not available
*C. albicans*			*C. albicans*	
*E. coli*	2	*E. coli*	*E. coli*	*E. coli*
*P. aeruginosa*			*P. aeruginosa*	
*E. coli*	3	*C. albicans*	*E. coli*	Not available
*P. aeruginosa*			*P. aeruginosa*	
*C. albicans*			*C. albicans*	

### Monomicrobial AST performance

Reference BMD testing was not performed on isolates that were considered off panel organisms by Pheno (*Bacteroides thetaiotaomicron n* = 1, *Candida* species *n* = 9, *Enterococcus gallinarum n* = 1, *Pseudomonas nitroreducens n* = 1, *Streptococcus* species *n* = 8 and *Staphylococcus caprae n* = 1)

The distributions of agreement and errors by individual antibiotics for each gram-positive and gram-negative organisms were analysed (Tables 4 and 5, respectively). In total, 99 monomicrobial cases were included in the final performance analysis comparing all 3 AST methods (Pheno, direct AST, colony AST) to BMD. Certain specimens failed to produce an AST on Pheno due to growth control failures or invalid results. Those isolates were: 3 *P. aeruginosa*, 1 *Enterobacter* spp., 1 *E. coli*, and 1 *E. faecalis*. Similarly, for direct Vitek AST, 3 *P. aeruginosa* and 1 coagulase negative *Staphylococcus* failed to provide AST due to growth control failure and low confidence.

**Table 4:**
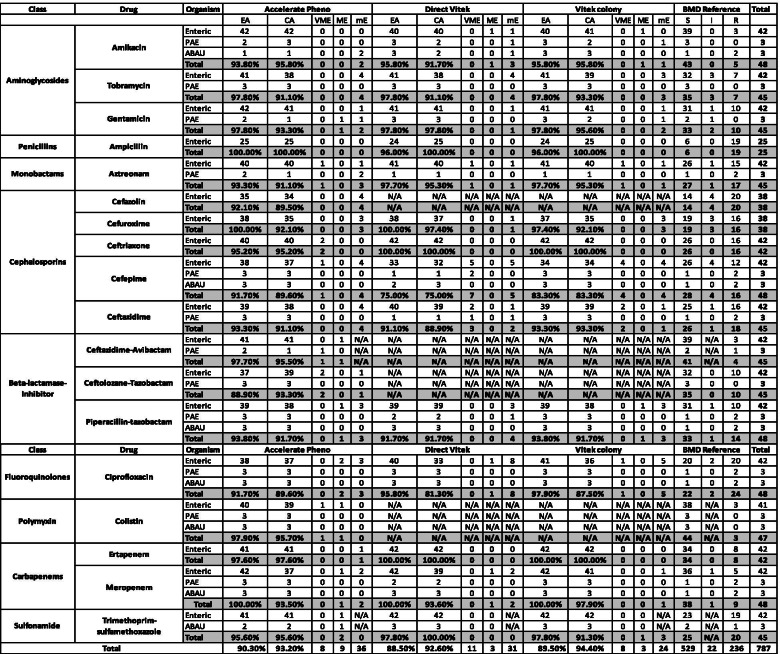
Monomicrobial gram negative AST performance

**Table 5:**
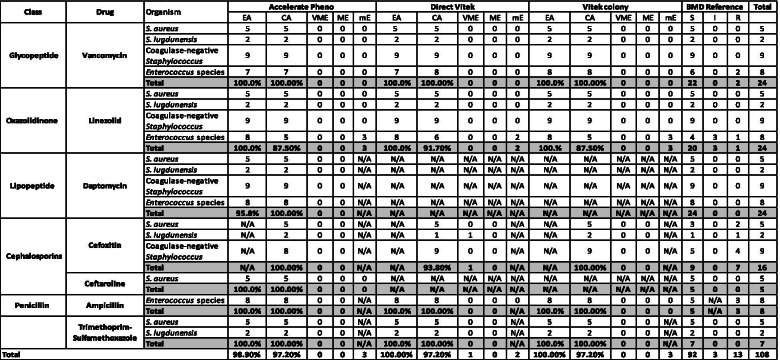
Monomicrobial gram positive AST performance

#### Resistance genotypes compared to phenotypic AST testing

Resistance as detected by phenotype and genotypic resistance mechanisms are compared in Table 6. There was 1 case where a methicillin resistant *S. epidermidis* (MR-CNS) was mixed with methicillin susceptible *S. aureus* (MSSA) and BCID2 correctly differentiated MSSA from MR-CNS by detecting *mecA/C* but not *mecA/C* plus MREJ, which is uniquely present in MRSA but absent from MR-CNS. There were 2 cases which *mecA* was detected in *S. hominis* in BCID while negative for *mecA/C* by BCID2. Both of isolates were also determined to be cefoxitin resistant on disk diffusion.

**Table 6:** Comparison of genotypic and phenotypic resistance detected for characterized isolates with known resistance genes and/or phenotypic profiles

Organism Group Characteristic	Total	BCID	BCID2	Direct AST	Pheno
***Staphylococcus*** **species**					
Methicillin-resistant *Staphylococcus aureus*	2	2/2 (mecA)	2/2 (mecA/C)	2/2 (cefoxitin)	2/2 (cefoxitin)
Methicillin-resistant coagulase-negative *Staphylococcus*	6	6/6	1/3	5/6 (cefoxitin)	5/6 ^a^ (cefoxitin)
***Enterococcus*** **species**					
Linezolid-resistant *Enterococcus*	2	N/A	N/A	2/2 (linezolid)	2/2 (linezolid)
Vancomycin-resistant *Enterococcus*	2	2/2 (van A/B)	2/2 (van A/B)	2/2 (vancomycin)	2/2 (vancomycin)
**Enterobacterales**					
Extended spectrum beta-lactamase disk test positive Enterobacterales	13	N/A	13/13 (CTX-M)	13/13 (ceftriaxone)	11/12^a^ (ceftriaxone)
Colistin-resistant Enterobacterales	1	N/A	1/1 (mcr-1)	N/A	1/1 (colistin)
Carbapenem-resistant, carbapenemase-positive (*bla*_KPC_) Enterobacterales	3	3/3 (KPC)	3/3 (KPC)	3/3 (meropenem R/ertapenem R)	3/3 (meropenem R/ertapenem R)
Carbapenem-susceptible, carbapenemase-positive (*bla*_KPC_) Enterobacterales	2	2/2 (KPC)	2/2 (KPC)	2/2 (ertapenem S, meropenem S)	2/2 ^b^ (ertapenem S)
Carbapenem-resistant Enterobacterales without resistant genes	5	N/A	N/A	5/5 ertapenem non susceptible	5/5 ertapenem non susceptible

^a^invalid result on Pheno

^b^meropenem suppressed

Carbapenem resistant Enterobacterales (CRE) that lacked detectable carbapenemase genes (as defined in the Methods section) were tested (*n* = 5). Of note, CRE at our institution is defined as either meropenem or ertapenem non susceptible (intermediate or resistant). Pheno and direct Vitek correctly identified all isolates as non-susceptible to ertapenem (5/5). Of the five isolates, there were 2 resistant, 1 intermediate and 2 susceptible to meropenem by BMD. Pheno identified four as resistant and 1 as intermediate, resulting in 1 major and 2 minor errors. Direct VITEK meropenem interpretations resulted in 1 major and 2 minor errors, with 1 minor error being intermediate by BMD and susceptible by direct VITEK. Both BCID and BCID2 were negative for carbapenemase genes for all 5 cases. Both BCID and BCID2 detected resistance genes in polymicrobial samples. For instance, BCID2 correctly identified the presence of *bla*_NDM_ present in *E. coli* in 2 polymicrobial samples, 1 of which was mixed with *P. aeruginosa* and the other mixed with *P. aeruginosa* and *C. albicans*. Another example is BCID2 correctly identified the presence of both *mecA/C* & MREJ and *vanA/B* genes in a case which a vancomycin-resistant *Enterococcus* (VRE) was mixed with MRSA.

Thirteen isolates positive for ESBL production by the CLSI ESBL disk test all tested positive for *bla*_CTX-M_. Of the 13 isolates, 13/13 were ceftriaxone-resistant by direct AST and 11/12 were resistant by Pheno (one isolate had invalid AST results). An additional 3 isolates were ceftriaxone resistant by direct AST and Pheno but likely had an ESBL other than *bla*_CTX-M_.

While some multi drug-resistant *P. aeruginosa* and *A. baumannii* isolates were included in this study, current molecular platforms do not detect resistant mechanisms that are predictive of antimicrobial resistance and thus they are not included in the genotypic-phenotypic performance analyses.

## Discussion

Rapid ID and AST of PBC has shown to greatly impact patient care and improve clinical outcomes [24]. To our knowledge, this is the first study in Asia evaluating the ID performance of Pheno and BCID2 directly from PBC bottles and the first study to evaluate the AST performance of Pheno and direct Vitek AST using a saponin-based extraction method.

The identification performance for Pheno, BCID and BCID2 observed in this data set was comparable to previously published performance data for identifications by other rapid methods such as short term culture with MALDI [4], BCID [25–29], Bruker Sepsityper [30], and GenMark ePlex [31]. The expanded panel of BCID2 provides additional target groups for clinically significant causative agents of bacteraemia; in particular, *B. fragilis* and *C. auris*. *B. fragilis* accounts for 45% of all anaerobic blood culture infections [32] and are attributed to a high mortality rate up to 25% [33]. Sepsis caused by multidrug-resistant *C. auris* is reported to be associated with high morbidity and mortality rates ranging from 30 to 72% [34], therefore timely identification is crucial for appropriate treatment initiation and infection control on the spread of multidrug-resistant *C. auris* in clinical settings. Furthermore, the addition of *mecA/C* & MREJ in BCID2 allows the differentiation between MRSA and MR-CNS in polymicrobial infections where these organisms are co-detected*.*

Despite BCID2 being an advanced version of BCID with expanded target groups and resistant genes, we observed a few notable differences in 2 samples when tested on the BCID compared with the BCID2. One sample of a *S. hominis* was *mecA*-positive when tested on the BCID platform but failed to detect the *mec*A/C gene on the BCID2. Additionally, a polymicrobial specimen containing *Proteus* species and *Enterococcus* species with the BCID, whereas BCID2 detected *Proteus* species only and failed to detect the presence of *Enterococcus* species, due to the replacement of the *Enterococcus* species target with species-specific probes for *E. faecalis* and *E. faecium* only*.*

With respect to AST, overall performance was comparable across the three AST methods evaluated in this study. A higher rate of VME was observed with direct Vitek (5.3% compared with 3.1 and 3.8% of Pheno and colony Vitek, respectively). This was largely attributed to more VMEs observed for cefepime and ceftazidime with direct Vitek. Some publications suggested that this high rate of VMEs might be due to the inoculum effect as the inoculum size of Vitek is smaller than with BMD [35]. The data observed here for cefepime also highlight the importance of considering use of a reference method, such as BMD, when evaluating a new AST assay, although this poses a practical challenge for clinical laboratories. Some published data sets evaluating direct Vitek have used Vitek from colony as a comparator method, in which case certain shortcomings of the assay are never identified such as would be the case for cefepime here if BMD was not performed [36].

It is important to note that the Advanced Expert System (AES) corrections by Vitek2 were ruled out in this study for true MIC comparisons between different methods, however in routine laboratory setting, AES was also applied for results interpretation. For instance, it is well published that MICs for certain β-lactam antibiotics with some Enterobacterales which produce ESBLs are found to be susceptible in vitro however clinical failures have been observed in vivo [37]. The additional testing of Vitek2 system on the ESBL and cefoxitin screens allowed the correction of false susceptibility on cephalosporins and oxacillin, respectively. With such supplementary interpretation systems, one mE and 15 VMEs were resolved by AES for *Enterobacteriaceae* and one VME was resolved in *Staphylococcus lugdunensis* in direct. Similarly, for colony Vitek, eight VMEs were resolved by AES. Despite the fact that some of the VMEs could be rectified by applying AES rules in Vitek, cefepime accounted for most of the MIC discrepancies in both direct and colony Vitek while in Pheno cefepime only accounted for a low level of MIC discrepancies. MIC discrepancies with cefepime are of concern as the evolving science of pharmacokinetics-pharmacodynamics has become increasingly important in recent years in determining MIC breakpoints and have even adopted a dose-dependent breakpoint [19]. This emphasises the importance of an accurate MIC value.

The invalid rate of all tested isolates was mainly attributed to mucoid *P. aeruginosa* strains for all three methods and occasionally occurred in Enterobacterales (*n* = 2) and *Enterococcus* (*n* = 1) for Pheno and coagulase-negative *Staphylococcus* species (*n* = 1) for direct AST. It is suggested that testing on mucoid strains is a known limitation for automated AST systems [38]. Due to this limitation, supplementary testing on mucoid strains by disk diffusion is recommended.

The performance of Pheno against gram positive organisms was excellent, with no major or very major errors observed and only 3 minor errors with linezolid against *Enterococcus* spp. A low level of mEs were observed in the testing of *Enterococcus* to linezolid for both direct (*n* = 2) and colony AST (*n* = 3) and one VME was noted in *S. lugdunensis* to cefoxitin for direct AST, however it could be resolved by applying AES corrections.

The organisms represented in this data set include most of the clinically important resistant genotypes and phenotypes in Hong Kong [39]. In addition, colistin is a crucial last resort drug choice for multidrug resistant microorganisms, however recent study has shown that the asymptomatic faecal carriage of *mcr-1*-harbouring *Enterobacteriaceae* was 2.08% [40]. The ability of BCID and BCID2 in rapid detection of antimicrobials resistance genes, in particular BCID2 provided additional clinical significant resistant genes targets such as *mecA/C* and MREJ, *bla*_VIM_ and *bla*_IMP_ *bla*_CTX-M,_ *bla*_NDM_ and *bla*_OXA-48_ and *mcr-*1 which allow clinicians to predict the antimicrobial resistant patterns and to execute appropriate and prompt treatment/ precautions. The overall concordance rate between resistant genes detected by BCID, BCID2 and phenotypic expressions detected by Pheno, direct and colony AST on most of the resistant genotypes/phenotypes was good. There were some isolates for which carbapenem resistance was not due to the presence of enzymes or the presence of CPE gene was not expressed phenotypically. This demonstrates the importance of both genotypic and phenotypic testing. While genotypic information is helpful to make predictions, phenotypic results may not always correlate according to the anticipated predictions.

Among the isolates with carbapenemase genes detected (*n* = 5), all were correctly identified and found to possess other ESBL genes (*bla*_CTX-M_ *n* = 9, *bla*_SHV_ *n* = 1, *bla*_TEM_ *n* = 1); some of these isolates were also resistant to quinolones and aminoglycosides. CREs that were correctly identified with the corresponding resistant genes were also found to have resistant carbapenem phenotypes for all 3 phenotypic detection methods. However, the resistance of antimicrobials cannot solely depend on genotypic detection. In Hong Kong, a local study in 2016 examined the clonality and mechanism of resistance of CRE isolates. It was shown that only 10% were genotypic carbapenemase-producing Enterobacterales (CPE) while porin loss combined with AmpC and/or CTX-M type ESBL was the major mechanism of resistance of the CREs [39].

For CRE isolates lacking the targeted carbapenemase genes in this study, Pheno correctly identified non susceptibility to ertapenem (5/5). Noteworthy, the low resistance detection rate could be accounted for the low number of tested samples, further studies for CREs lacking carbapenemase genes would be recommended.

Conversely, detection of carbapenemase genes may not always confer an antimicrobial resistance phenotype, for isolates with carbapenemase genes without phenotypic expressions; both BCID and BCID2 correctly detected the presence of carbapenemase genes. Pheno correctly identified ertapenem as susceptible while for meropenem, all cases (*n* = 2) were failed to provide results, due to inconclusive assay results. For both direct and colony AST, the susceptibility of ertapenem and meropenem was correctly identified. Since genotypic and phenotypic expression of resistant genes do not always co-exist and correspond, therefore the adoption of both BCID/BCID2 (genotypic) in combination with direct AST (phenotypic) methods can allow clinicians to predict the effectiveness of antimicrobials in an accurate and timely manner. Furthermore, other clinical significant antimicrobial resistant microorganisms such as linezolid non susceptible *Enterococcus* species and multi drug-resistant *A. baumannii*, which resistant genes are not available on BCID and BCID2 detection panel, Pheno, direct AST and colony AST demonstrated satisfactory performance with low level of minor errors (Pheno *n* = 2, direct AST *n* = 1, colony AST *n* = 2).

Antimicrobial treatment choices for sepsis are often empirical and based on the susceptibility profile of the most common causative agents for sepsis. However, the causative agents profile may vary slightly depending on the institution and local epidemiology. The most commonly found causative agents for sepsis in 2019 at our institution were *E. coli* (32.9%)*, K. pneumoniae* (6.9%)*, S. aureus* (5.8%)*, E. faecalis* (4.3%)*, P. aeruginosa* (3.2%) and Coagulase-negative *Staphylococcus* (2.9%). The Accelerate PhenoTest® BC kit used in this evaluation includes 17 ID organisms covering the majority of these causative agents and 7 g-positive and 19 g-negative antimicrobials. In comparison, BCID provides additional 10 ID targets (totalling 27), which include *H. influenzae, N. meningitides, L. monocytogenes*, *C. krusei, C. parapsilosis*, *C. tropicalis* and species-level identification for organisms that are limited to genus level detection in Pheno, such as: *K. oxytoca, K. pneumoniae, S. agalactiae, S. pneumoniae,* and *S. pyogenes,* but only 3 resistance genes. BCID2 expands on BCID with an additional 5 ID and 7 resistance gene targets. The lack of species-differentiation of Pheno is not a major concern for most Enterobacterales because the breakpoints in CLSI M100 for Enterobacterales are classified in the same group. Nevertheless, for some organisms, identification to species level is of clinical significance, in particular *Streptococcus* species. It is critical to differentiate clinically significant species such as *S. pneumoniae* and *S. pyogenes* from those that are typically considered as contaminants, like Viridans-group *Streptococcus* species and other beta-hemolytic *Streptococcus* species. The rate for these groups of organisms at our institution in 2019 was only 0.4 and 0.7%, respectively.

On the other hand, Vitek panel provides a wider range of antimicrobial panel choice than Pheno for both gram negative and gram-positive microorganisms. Both Pheno and Vitek have novel antimicrobials such as ceftazidime-avibactam and ceftolozane-tazobactam on their panels, however, the newer Vitek card with these antimicrobials was not evaluated in this study. Studies have demonstrated that the usage of ceftazidime-avibactam and ceftolozane-tazobactam had significant improvement in activity against Enterobacterales with EBSLs and multi drug-resistant *P. aeruginosa*. Therefore, the availability of these novel antimicrobials on diagnostics devices could provide additional drug choice for clinicians when treating patients with these more complicated infections [41, 42].

An integrated ID and AST system such as Pheno allows for easy workflow and reduced time to result, however such integrated system might lead to another issue when interpreting both results in combination. There was 1 case in which *E. gallinarum* was incorrectly identified as *E. faecium* but was able to provide AST result. This misidentification could lead to inappropriate prescription of vancomycin since all *E. gallinarum* exhibit low level vancomycin resistance due to the presence of *vanC* gene. For this case, Pheno did demonstrate intermediate resistance for vancomycin but nonetheless, was still an incorrect ID.

On average, the time to result for PBC using conventional colony-based method is around 48–72 h due to the requirement of overnight incubation for colony ID and AST. Both (1) Pheno and (2) BCID/BCID2 and direct AST have significantly reduced the time to result compared to conventional colony based ID and AST: the TAT for Pheno was around 7 h (90 mins for ID and 7 h for AST) with 2 min hands-on time; whereas for BCID/BCID2 with direct AST, the total TAT was around 9 to 20 h (1 h for ID and 9–19 h for AST), with hands-on time of 15 min. Less hands-on time eased our laboratory technicians’ labour and streamlined workflow. Furthermore, faster TAT enabled our physicians to make faster and accurate clinical decisions [43]. Though cost effectiveness analysis is not performed in this study, it is believed that the improvement in TAT for ID/ AST would translate into better clinical outcome such as optimal use of antimicrobials, fewer complications, shorter hospital stay, etc. These would in return result in a more cost effective management for PBC cases [5]. Additional studies to look into the cost effectiveness in shortening the TAT for ID/ AST would be important for driving the change in practices.

Although empirical antibiotics are often given prior to the availability of ID and AST results, several studies have shown that incorrect use of antibiotics even for a short duration could associate with increased risk of acute kidney injury [44]. Therefore, rapid and accurate ID and AST results could reduce unnecessary exposure to drug toxicity and antimicrobial resistance.

In conclusion, both 1) Pheno and 2) BCID/BCID2 with direct AST methods achieved satisfactory ID and AST results. The direct Vitek AST method achieved good and comparable antimicrobial susceptibility performance with conventional colony Vitek AST. Although several studies have been published to evaluate the performance of positive blood direct extraction for ID however, the procedure and equipment required were more complicated and none were published on evaluating direct AST with the same sample preparation [13–18, 45–54]. With the direct Vitek method used in this study, along with the accurate ID and resistance genes included on the BCID and BCID2 panels, this integrated method can provide fairly rapid and accurate results. To implement proper methods, clinical laboratories should consider and evaluate different methodologies in order to select the best method to fit their routine workflow. Nevertheless, there are limitations of our study in that for some species, very few isolates were represented in this data set e.g. *Enterobacter* species (*n* = 1), *Serratia marcescens* (*n* = 1) and *S. lugdunensis* (*n* = 2). Therefore, further studies would be recommended for optimal determination of system performance on both ID and AST [43]. This study was also limited by the small numbers of fresh clinical samples. However, it has already demonstrated the accuracy and advantages of the various methods.

Although all these rapid methods demonstrated promising ID and AST results, the practice of gram-stain and sub-culturing of PBC would still be useful and important as these rapid methods have limitations, such as detecting and/or performing AST on polymicrobial samples, performing AST on mucoid strains such as *P. aeruginosa*, a known limitation for automated systems when performing colony AST [38]. Furthermore, some commercial platforms such as Verigene or ePlex require a prior gram stain result to facilitate the selection of the panel.

In laboratories where non daytime working hours were staffed by staff with limited training or not proficient in microbiology, the ease of lean workflow and result interpretations of Pheno are advantageous in such a setting with fewer technician interventions and techniques required, whereas despite the significant reduction in hands-on time for BCID/BCID2 with direct AST compared to conventional methods, the operation of direct AST still required highly trained microbiology staff and intervention time. Pheno can easily be set up around the clock whenever a PBC is available. Both workflows evaluated in this study provide different solutions for laboratories looking for more direct ID and AST methods but must be considered individually for each institution’s needs and practices.

## Supplementary Information

**Additional file 1.**

## Data Availability

The datasets used and/or analysed during the current study available from the corresponding author on reasonable request. The datasets are available in the figshare repository. 10.6084/m9.figshare.14750685.
