# Supplementary material for: Comparison of novel rapid diagnostic of blood culture identification and antimicrobial susceptibility testing by Accelerate Pheno system and BioFire FilmArray Blood Culture Identification and BioFire FilmArray Blood Culture Identification 2 panels
Source: BMC Microbiol. 2021 Dec 18;21:350. doi: 10.1186/s12866-021-02403-y (PMC8684256; doi:10.1186/s12866-021-02403-y)
Supplement: Supplementary file 1 — Additional file 1. [file 12866_2021_2403_MOESM1_ESM.docx]

Supplementary table

|  | | | **ID** | | | **AST** | | |
| --- | --- | --- | --- | --- | --- | --- | --- | --- |
| **Sample** | **Sample types** | **Number of organism(s)** | **Pheno** | **BCID** | **BCID2** | **Pheno AST** | **Direct AST** | **Colony AST** |
| sample 001 | fresh | 1 | Y | Y | N | Y | N | Y |
| sample 002 | seeded | 2 | Y | Y | N | N | N | N |
| sample 003 | fresh | 1 | Y | Y | N | Y | N | Y |
| sample 004 | fresh | 1 | Y | Y | N | Y | Y | Y |
| sample 005 | fresh | 1 | Y | Y | Y | Y | Y | Y |
| sample 006 | fresh | 1 | Y | Y | N | Y | Y | Y |
| sample 007 | seeded | 1 | Y | Y | N | Y | Y | Y |
| sample 008 | fresh | 1 | Y | Y | N | Y | Y | Y |
| sample 009 | fresh | 1 | Y | Y | N | Y | Y | Y |
| sample 010 | fresh | 1 | Y | Y | N | Y | Y | Y |
| sample 011 | fresh | 1 | Y | Y | N | Y | Y | Y |
| sample 012 | seeded | 1 | Y | Y | N | Y | Y | Y |
| sample 013 | seeded | 1 | Y | Y | Y | Y | Y | Y |
| sample 014 | fresh | 1 | Y | Y | N | Y | Y | Y |
| sample 015 | fresh | 1 | Y | Y | N | N | N | N |
| sample 016 | seeded | 1 | Y | Y | Y | Y | Y | Y |
| sample 017 | seeded | 1 | Y | Y | Y | Y | Y | Y |
| sample 018 | seeded | 1 | Y | Y | Y | Y | Y | Y |
| sample 019 | fresh | 1 | Y | Y | Y | Y | Y | Y |
| sample 020 | fresh | 1 | Y | Y | N | Y | Y | Y |
| sample 021 | fresh | 1 | Y | Y | N | Y | Y | Y |
| sample 022 | fresh | 1 | Y | Y | N | Y | Y | Y |
| sample 023 | fresh | 1 | Y | Y | N | Y | Y | Y |
| sample 024 | fresh | 1 | Y | Y | N | Y | Y | Y |
| sample 025 | fresh | 1 | Y | Y | Y | Y | Y | Y |
| sample 026 | fresh | 1 | Y | Y | N | Y | Y | Y |
| sample 027 | fresh | 1 | Y | Y | Y | Y | Y | Y |
| sample 028 | fresh | 1 | Y | Y | Y | Y | Y | Y |
| sample 029 | fresh | 1 | Y | Y | Y | Y | Y | Y |
| sample 030 | fresh | 3 | Y | Y | Y | N | N | N |
| sample 031 | fresh | 2 | Y | Y | Y | N | N | N |
| sample 032 | fresh | 1 | Y | Y | N | Y | Y | Y |
| sample 033 | fresh | 1 | Y | Y | N | Y | Y | Y |
| sample 034 | fresh | 1 | Y | Y | Y | Y | Y | Y |
| sample 035 | fresh | 1 | Y | Y | N | Y | Y | Y |
| sample 036 | fresh | 1 | Y | Y | Y | Y | Y | Y |
| sample 037 | fresh | 1 | Y | Y | Y | Y | Y | Y |
| sample 038 | fresh | 1 | Y | Y | N | Y | Y | Y |
| sample 039 | fresh | 1 | Y | Y | Y | Y | Y | Y |
| sample 040 | fresh | 1 | Y | Y | N | Y | Y | Y |
| sample 041 | fresh | 1 | Y | Y | N | Y | Y | Y |
| sample 042 | fresh | 1 | Y | Y | N | Y | Y | Y |
| sample 043 | fresh | 1 | Y | Y | N | Y | Y | Y |
| sample 044 | fresh | 1 | Y | Y | N | Y | Y | Y |
| sample 045 | fresh | 1 | Y | Y | N | Y | Y | Y |
| sample 046 | fresh | 1 | Y | Y | Y | Y | Y | Y |
| sample 047 | fresh | 1 | Y | Y | N | Y | Y | Y |
| sample 048 | fresh | 1 | Y | Y | N | Y | Y | Y |
| sample 049 | fresh | 1 | Y | Y | N | Y | Y | Y |
| sample 050 | fresh | 1 | Y | Y | N | Y | Y | Y |
| sample 051 | fresh | 1 | Y | Y | N | Y | Y | Y |
| sample 052 | fresh | 1 | Y | Y | N | Y | Y | Y |
| sample 053 | fresh | 1 | Y | Y | N | Y | Y | Y |
| sample 054 | fresh | 1 | Y | Y | N | Y | Y | Y |
| sample 055 | fresh | 1 | Y | Y | N | Y | Y | Y |
| sample 056 | fresh | 2 | Y | Y | Y | N | N | N |
| sample 057 | fresh | 1 | Y | Y | N | Y | Y | Y |
| sample 058 | fresh | 1 | Y | Y | N | Y | Y | Y |
| sample 059 | fresh | 1 | Y | Y | N | Y | Y | Y |
| sample 060 | fresh | 1 | Y | Y | N | Y | Y | Y |
| sample 061 | fresh | 1 | Y | Y | N | Y | Y | Y |
| sample 062 | fresh | 1 | Y | Y | N | Y | Y | Y |
| sample 063 | fresh | 1 | Y | Y | N | Y | Y | Y |
| sample 064 | fresh | 1 | Y | Y | N | Y | Y | Y |
| sample 065 | fresh | 1 | Y | Y | N | Y | Y | Y |
| sample 066 | fresh | 1 | Y | Y | N | Y | Y | Y |
| sample 067 | fresh | 1 | Y | Y | Y | Y | Y | Y |
| sample 068 | seeded | 1 | Y | Y | N | Y | Y | Y |
| sample 069 | seeded | 1 | Y | Y | Y | Y | Y | Y |
| sample 070 | fresh | 1 | Y | Y | N | Y | Y | Y |
| sample 071 | seeded | 1 | Y | Y | N | Y | Y | Y |
| sample 072 | seeded | 1 | Y | Y | Y | Y | Y | Y |
| sample 073 | fresh | 1 | Y | Y | N | Y | Y | Y |
| sample 074 | seeded | 1 | Y | Y | Y | Y | Y | Y |
| sample 075 | seeded | 1 | Y | Y | Y | Y | Y | Y |
| sample 076 | seeded | 1 | Y | Y | Y | Y | Y | Y |
| sample 077 | fresh | 1 | Y | Y | N | Y | Y | Y |
| sample 078 | fresh | 1 | Y | Y | N | Y | Y | Y |
| sample 079 | seeded | 2 | Y | Y | Y | Y | Y | Y |
| sample 080 | seeded | 1 | Y | Y | Y | Y | Y | Y |
| sample 081 | fresh | 1 | Y | Y | N | Y | Y | Y |
| sample 082 | fresh | 1 | Y | Y | N | Y | Y | Y |
| sample 083 | fresh | 1 | Y | Y | N | Y | Y | Y |
| sample 084 | seeded | 1 | Y | Y | Y | Y | Y | Y |
| sample 085 | seeded | 1 | Y | Y | N | Y | Y | Y |
| sample 086 | seeded | 1 | Y | Y | Y | Y | Y | Y |
| sample 087 | seeded | 1 | Y | Y | N | Y | Y | Y |
| sample 088 | fresh | 1 | Y | Y | N | Y | Y | Y |
| sample 089 | fresh | 1 | Y | Y | N | Y | Y | Y |
| sample 090 | seeded | 1 | Y | Y | Y | Y | Y | Y |
| sample 091 | seeded | 1 | Y | Y | N | Y | Y | Y |
| sample 092 | seeded | 1 | Y | Y | Y | Y | Y | Y |
| sample 093 | seeded | 1 | Y | Y | Y | Y | Y | Y |
| sample 094 | seeded | 1 | Y | Y | Y | Y | Y | Y |
| sample 095 | fresh | 1 | Y | Y | N | Y | Y | Y |
| sample 096 | fresh | 1 | Y | Y | N | Y | Y | Y |
| sample 097 | seeded | 1 | Y | Y | N | Y | Y | Y |
| sample 098 | seeded | 1 | Y | Y | Y | Y | Y | Y |
| sample 099 | seeded | 1 | Y | Y | N | Y | Y | Y |
| sample 100 | fresh | 1 | Y | Y | N | Y | Y | Y |
| sample 101 | seeded | 2 | Y | Y | N | N | N | N |
| sample 102 | seeded | 2 | Y | Y | Y | N | N | N |
| sample 103 | seeded | 1 | Y | Y | N | Y | Y | Y |
| sample 104 | seeded | 1 | Y | Y | N | Y | Y | Y |
| sample 105 | seeded | 1 | Y | Y | N | Y | Y | Y |
| sample 106 | seeded | 1 | Y | Y | N | Y | Y | Y |
| sample 107 | seeded | 2 | Y | Y | Y | N | N | N |
| sample 108 | seeded | 2 | Y | Y | Y | N | N | N |
| sample 109 | seeded | 1 | Y | Y | N | Y | Y | Y |
| sample 110 | seeded | 1 | Y | Y | Y | Y | Y | Y |
| sample 111 | seeded | 2 | Y | Y | Y | N | N | N |
| sample 112 | seeded | 2 | Y | Y | N | N | N | N |
| sample 113 | seeded | 2 | Y | Y | Y | N | N | N |
| sample 114 | seeded | 2 | Y | Y | Y | N | N | N |
| sample 115 | seeded | 2 | Y | Y | Y | N | N | N |
| sample 116 | seeded | 3 | Y | Y | Y | N | N | N |
| sample 117 | seeded | 1 | N | N | Y | N | N | N |
| sample 118 | seeded | 1 | N | N | Y | N | N | N |
| sample 119 | seeded | 1 | N | N | Y | N | N | N |
| sample 120 | seeded | 1 | N | N | Y | N | N | N |
| sample 121 | seeded | 3 | N | N | Y | N | N | N |
| sample 122 | seeded | 1 | N | N | Y | N | N | N |
| sample 123 | seeded | 1 | N | N | Y | N | N | N |
| sample 124 | seeded | 1 | N | N | Y | N | N | N |
| sample 125 | seeded | 0 | N | N | Y | N | N | N |
| sample 126 | seeded | 3 | N | N | Y | N | N | N |
| sample 127 | seeded | 1 | N | N | Y | N | N | N |
| sample 128 | seeded | 1 | N | N | Y | N | N | N |
| sample 129 | seeded | 1 | N | N | Y | N | N | N |
| sample 130 | seeded | 1 | N | N | Y | N | N | N |
| sample 131 | seeded | 1 | N | N | Y | N | N | N |
| sample 132 | seeded | 2 | N | N | Y | N | N | N |
| sample 133 | seeded | 1 | N | N | Y | N | N | N |
| sample 134 | seeded | 2 | N | N | Y | N | N | N |
| sample 135 | seeded | 3 | N | N | Y | N | N | N |
| **Total number of samples tested** | | | 116 | 116 | 56 | 101 | 99 | 101 |

Y = Yes

N = No
